# Characterization of a TiO_2_ enrichment method for label-free quantitative phosphoproteomics

**DOI:** 10.1016/j.ymeth.2011.02.004

**Published:** 2011-08

**Authors:** Alex Montoya, Luisa Beltran, Pedro Casado, Juan-Carlos Rodríguez-Prados, Pedro R. Cutillas

**Affiliations:** Analytical Signalling Laboratory, Centre for Cell Signalling, Barts Cancer Institute, Bart’s and the London School of Medicine, Queen Mary University of London, Charterhouse Square, London EC1M 6BQ, UK

**Keywords:** Acc, accuracy, ACN, acetonitrile, AmAc, ammonium acetate, CV, coefficient of variation, IMAC, immobilized metal affinity chromatography, LC–MS/MS, liquid chromatography tandem mass spectrometry, MS, mass spectrometry, SCX, strong cation exchange, TiO_2_, titanium dioxide, XIC, extracted ion chromatogram, Biomarker, Cell signalling, Kinase, Label-free, LC-MS/MS, Mass spectrometry, Phosphorylation, Phosphoproteomics, Quantitative analysis, Systems biology, Titanium dioxide

## Abstract

Phosphorylation is a protein post-translational modification with key roles in the regulation of cell biochemistry and signaling. In-depth analysis of phosphorylation using mass spectrometry is permitting the investigation of processes controlled by phosphorylation at the system level. A critical step of these phosphoproteomics methods involves the isolation of phosphorylated peptides from the more abundant unmodified peptides produced by the digestion of cell lysates. Although different techniques to enrich for phosphopeptides have been reported, there are limited data on their suitability for direct quantitative analysis by MS. Here we report a TiO_2_ based enrichment method compatible with large-scale and label-free quantitative analysis by LC–MS/MS. Starting with just 500 μg of protein, the technique reproducibly isolated hundreds of peptides, >85% of which were phosphorylated. These results were obtained by using relatively short LC–MS/MS gradient runs (45 min) and without any previous separation step. In order to characterize the performance of the method for quantitative analyses, we employed label-free LC–MS/MS using extracted ion chromatograms as the quantitative readout. After normalization, phosphopeptides were quantified with good precision (coefficient of variation was 20% on average, *n* = 900 phosphopeptides), linearity (correlation coefficients >0.98) and accuracy (deviations <20%). Thus, phosphopeptide ion signals correlated with the concentration of the respective phosphopeptide in samples, making the approach suitable for in-depth relative quantification of phosphorylation by label-free LC–MS/MS.

## 1 Introduction

Protein phosphorylation is a key process in the regulation of cell homeostasis [1]. In addition, diseases of pressing importance in the developed world, such as diabetes, neurodegeneration and cancer, are the result of processes controlled by protein phosphorylation [2–6]. This post-translational modification is controlled by two families of proteins: kinases and phosphatases, which add or remove the phosphate group, respectively. Thus, study of the phosphoproteome allows us to infer the activation status of kinase/phosphatase reaction pairs with unprecedented depth [7]. This is in turn paving the way for a better understating of cell biochemistry at the system level.

Although fully occupied sites exist, most phosphorylated proteins are present at sub-stoichiometric levels relative to their unphosphorylated counterparts. Thus, techniques that allow enriching samples for phosphorylated proteins (or phosphopeptides after protease digestion) before mass spectrometry (MS) analysis are needed for the success of phosphoproteomics approaches [8]. Strong cation exchange chromatography (SCX) [9], immunoprecipitation [10], immobilized metal ion affinity chromatography (IMAC) [11,12], titanium dioxide affinity purification (TiO_2_) [13] and calcium precipitation [14], have been reported, among other techniques, to successfully isolate phosphopeptides and phosphoproteins prior to MS analysis. Liquid Chromatography coupled to tandem MS (LC–MS/MS) represents a suitable platform for large-scale phosphoproteomics, allowing the identification and quantification of thousands of phosphorylation sites per run [15,16].

TiO_2_ has proven to be particularly useful for phosphopeptide enrichment prior to LC–MS/MS analysis. However, although a large body of work has demonstrated the utility of this technique for phosphopeptide enrichment [17–19], there is limited data documenting the performance of TiO_2_ extraction for direct quantitative analysis. This is particularly important for quantitative approaches that do not rely on metabolic labeling, such as those based on chemical labeling and label-free quantification. In chemical labeling approaches, such as iTRAQ, phosphopeptides are labeled and mixed after their isolation by TiO_2_ or other methods [20], thus putting demands on the reproducibility of the isolation technique. The reproducibility of sample handling processes also contributes to the accuracy of label-free methods. Quantification by label-free techniques is attractive because these can be used to compare large sample numbers and primary samples (needed for robust statistics and clinical studies). The aim of the present study was to investigate the suitability and performance of a TiO_2_ chromatographic method to extract phosphopeptides in a form compatible with their analysis by direct label-free LC–MS/MS.

**Fig. 1:**
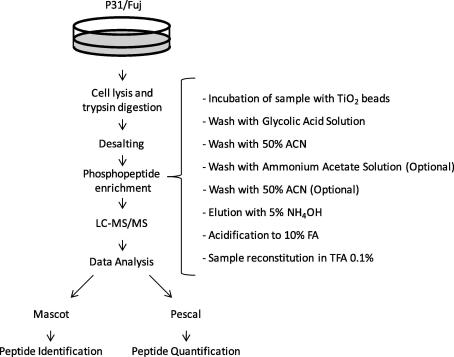
Workflow of TiO_2_ based phosphopeptide enrichment used in this study. Samples are lyzed, reduced, alkylated, digested with trypsin and desalted by RP SPE. The protocol for TiO_2_ enrichment includes the binding of peptides to TiO_2_ beads followed by the removal of unphosphorylated peptides by sequential washing with glycolic acid solution, 50% ACN, ammonium acetate solution (optional) and 50% ACN (optional). Phosphopeptides are then eluted using NH_4_OH, acidified, dried in a SpeedVac and, after reconstitution, analyzed by LC–MS/MS.

The present protocol (Fig. 1) involves the digestion of cellular proteins with trypsin and the removal of interfering salts and other small molecules prior to TiO_2_ phosphopeptide-enrichment and LC–MS/MS analysis. The optimized technique enabled robust and reproducible isolation of hundreds of phosphopeptides from small amounts of biological material. Importantly, our experiments show that the technique can be used to quantify phosphorylation in relatively short times (without the need for multidimensional chromatography) with precision, linearity and accuracy.

## 2 Methods

### 2.1 Cell culture

The acute myeloid leukemia (AML) cell line P31/Fuj was grown in RPMI-1640 medium supplemented with 10% FBS, 100 units/mL of Penicillin/Streptomycin and 50 μM β-Mercaptoethanol at 37 °C in a humidified atmosphere at 5% CO_2_. Cells were maintained at about 0.5–2 × 10^6^ cells/mL. Twenty-four hours prior to harvest, 50 × 10^6^ cells were seeded at a density of 0.5 × 10^6^ cells/mL in fresh medium. For linearity and accuracy assessment, cells were treated with vehicle (Control) or 1 mM sodium pervanadate (pV) for 30 min. Pervanadate was prepared by mixing 30% H_2_O_2_ and 100 mM Na_3_VO_4_ pH 8.0 at 1:100 ratio.

### 2.2 Cell lysis and trypsin digestion

Cell lysis and digestion was carried out as described [15]. Briefly, cells were collected by centrifugation at 300*g* for 5 min, washed twice with ice cold PBS supplemented with phosphatase inhibitors (1 mM Na_3_VO_4_ and 1 mM NaF) and lysed with a denaturing buffer (20 mM HEPES pH 8.0, 8 M urea, 1 mM Na_3_VO_4_, 1 mM NaF, 2.5 mM Na_4_P_2_O_7_, 1 mM ß-glycerol-phosphate) at a concentration of 10 × 10^6^ cells/mL. Cell lysates were further homogenized by sonication and insoluble material was removed by centrifugation at 20,000*g* for 10 min. Protein concentration in the supernatants was calculated by Bradford analysis and for each sample 0.5 mg of protein were resuspended in a volume of 1 mL of denaturing buffer. For linearity and accuracy assessment, control and treated cell lysates were mixed to a final protein concentration of 0.5 mg/mL. The proportions used were 0%, 25%, 50%, 75% and 100% of (pV) treated extracts mixed with 100%, 75%, 50%, 25% and 0% of vehicle treated extracts, respectively. For reduction and alkylation, protein mixtures were sequentially incubated with 4.1 mM DTT and 8.3 mM iodoacetamide for 15 min. For digestion, samples were diluted to 2 M urea with 20 nM HEPES pH 8.0 and incubated with immobilized TLCK-trypsin (20 TAME units/mg) for 16 h at 37 °C. Digestion was stopped by addition of TFA at a final concentration of 1%.

### 2.3 Desalting

The resultant peptide solutions were desalted by solid phase extraction (SPE) using Oasis HLB extraction cartridges (Waters UK Ltd., Manchester, UK) according to manufacturer instructions with some modifications. Briefly, cartridges were activated with 1 mL of 100% ACN and equilibrated with 1.5 mL of wash solution (2% ACN, 0.1% TFA in water). After the cartridges were loaded with peptide solution, they were washed with 1 mL of wash solution. Peptides were eluted with 0.5 mL of glycolic acid solution (1 M Glycolic acid in 80% ACN and 5% TFA). All the steps were done in a vacuum manifold set at 5 mm Hg.

### 2.4 TiO_2_ phosphoenrichment

Phosphopeptide enrichment was performed using a TiO_2_ protocol adapted for label free quantitative proteomics. In short, eluates from Oasis cartridges were normalized to 1 mL with glycolic acid solution and incubated for the indicated times (see Results) at room temperature with varying volumes of TiO_2_ solution (50% slurry, GL Sciences Inc., Japan). TiO_2_ beads were then packed by centrifugation in equilibrated C-18 spin columns (PepClean C-18 Spin Columns, Thermo Scientific, Rockford, IL). Beads were sequentially washed with 300 μL of glycolic acid solution, 50% ACN and ammonium acetate solution (20 mM ammonium acetate pH 6.8 in 50% ACN). An extra 50% ACN wash can be also added after the ammonium acetate solution. For phosphopeptide elution, beads were incubated three times with 50 μL 5% NH_4_OH for 1 min at room temperature and centrifuged. The three eluates of each fraction were pooled and acidified by addition of FA to a final concentration of 10%. Samples were then dried using a SpeedVac and pellets were stored at −80 °C.

### 2.5 Nanoflow-liquid chromatography tandem mass spectrometry (LC–MS/MS)

LC-MS/MS analysis was performed as described in [15]. In brief, phosphopeptide pellets were dissolved in 10–20 μl of 0.1% TFA and run in a LTQ-Orbitrap XL mass spectrometer (Thermo Fisher Scientific, Hemel Hempstead, UK) coupled online to a nanoflow ultra-high pressure liquid chromatography (UPLC, nanoAcquity, Waters). The UPLC settings consisted of a loading flow rate of 2 μL/min for 8 min followed by a gradient elution of 400 nL/min with an operating back pressure of about 3000 psi. Peptide separations were performed in a 100 μm × 100 mm column (BEH130 C18, 1.7 μm Waters) using solution A (0.1% FA in LC–MS grade water) and solution B (0.1% FA in LC–MS grade ACN) as mobile phases. Gradient runs were from 1% to 35% B in 45 min followed by a 5 min wash at 85% B and a 10 min equilibration step at 1% B. For some experiments, ACN gradient times were modified to 25, 50, 100 and 150 min. Full scan survey spectra (*m*/*z* 375–1800) were acquired in the Orbitrap with a resolution of 30000 at *m*/*z* 400. A data dependent analysis (DDA) was employed in which the five most abundant multiply charged ions present in the survey spectrum were automatically mass-selected, fragmented by collision-induced dissociation (normalized collision energy 35%) and analyzed in the LTQ. Thus, a maximum of five MS/MS scans (*m*/*z* 50–2000) were performed after each Full MS Scan resulting in a maximum duty cycle of 2.5 s. Dynamic exclusion was enabled with the exclusion list restricted to 500 entries, exclusion duration of 40 s and mass window of 10 ppm. Since chromatographic peaks were about 30 s at the base, these settings ensured that there were at least 10 data points per extracted ion chromatogram (XIC).

### 2.6 MS data analysis

Mascot Daemon (v2.2.2; Matrix Science, London, UK) was used to analyze the MS data. This software automates the use of Mascot Distiller 2.3.2., to smooth and centroid the MS/MS data, and Mascot search engine, to search the processed files against the peptide sequence library in the Swiss Prot database restricted to Human entries (version 2010_03 containing 23,000 entries, http://expasy.org/sprot/). The parameters included; restriction to human taxonomy, trypsin as digestion enzyme with up to two missed cleavages permitted, carbamidomethyl (C) as a fixed modification and Pyro-glu (*N*-term), Oxidation (M) and Phospho (STY) as variable modifications. Datasets were searched with a mass tolerance of ±7 ppm and a fragment mass tolerance of ±600 mmu. Hits were considered significant when they had an Expectation value <0.05 (as returned by Mascot). False discovery rates were ∼2% as determined by decoy database searches.

An in-house script was used to extract Mascot results, which were then placed in Excel files for further analysis. For peptides with multiple potential phosphorylation sites, the delta score between the first and second hits reported by Mascot was used to identify the correct position [21].

Pescal [22] was used to automate the generation of extracted ion chromatograms (XIC) and to calculate the peak heights and areas. Because of undersampling and the stochastic nature of peak selection for fragmentation in DDA experiments, MS/MS data was not obtained for all the phosphopeptides in all the runs. To overcome this issue, phosphopeptides identified by Mascot with a statistical value above the significance threshold were annotated in a database that includes their molecular mass, charge, retention time (tR) and sequence. The intensity of the phosphopeptides included in the database was quantified using Pescal. XICs were constructed for the first three isotopes of each peptide ion. This allows the application of restrictions on the *m*/*z*, tR, charge and isotope distribution. Windows for XIC construction were 7 ppm for *m*/*z*, 5 min for tR and a coefficient of correlation >0.95 between the observed and expected isotope distribution. The intensity values could then be calculated by determining the peak height and areas of each individual XIC. The resulting quantitative data were parsed into Excel files for normalization and statistical analysis. Peptide intensities were normalized to the total chromatogram intensity and further expressed as a percentage relative to the largest intensity value across samples.

### 2.7 Statistical analysis

Precision of quantification was expressed as the coefficient of variation (CV) of replicate measurements, which was calculated as the percentage of the ratio between the standard deviation and the mean. Linearity and accuracy of quantification were calculated as described in [15]. Briefly, to assess linearity, Excel was used to calculate Pearson’s correlation coefficient (*R*^2^). Since linear regression functions were constructed using five data points, correlations were considered statistically significant if *R*^2^ > 0.878 (*p* < 0.05). To assess accuracy, linear regression functions between phosphopeptide ion intensities and percentage of cell extract in the protein mixture were used to determine the theoretical relative peptide intensities. The deviation from total accuracy for each phosphopeptide was calculated by subtracting the observed relative intensity of the phosphopeptide from the theoretical intensity, dividing this value by the relative intensity and multiplying it by 100. The mean percentage accuracy (%Acc) was then calculated by averaging the accuracies of all the data points from each dilution.

## 3 Results

### 3.1 Efficiency of phosphopeptide enrichment

**Fig. 2:**
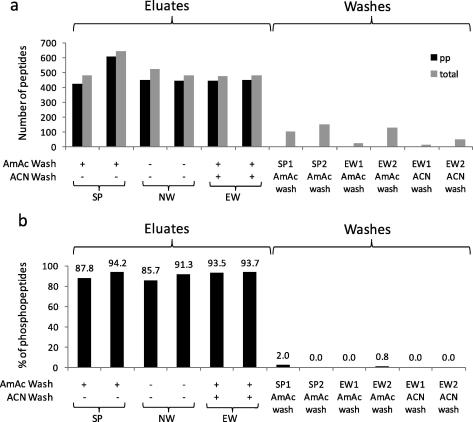
Assessment of the effects of different combinations of washes on the number of identified peptides and enrichment efficiency. Samples were enriched for phosphopeptides as in Fig. 1. Three different protocols were used: a standard protocol (SP) that does not include the second wash with 50% ACN (Fig. 1); a no wash protocol (NW) that does not include either the wash with ammonium acetate or the second ACN wash; and an extra wash protocol (EW) that includes both washes. Eluates and washes were then analyzed by LC–MS/MS. Peptides and phosphopeptides returned by Mascot with expectancy scores <0.05 were recorded. Each protocol was repeated twice. (a) Number of total peptides and phosphopeptides identified in the eluates and in the washes. (b) Percentage of phosphopeptides relative to total number of peptides in the eluates and washes.

We first evaluated the efficiency of our TiO_2_ protocol as a method to enrich phosphopeptides from complex mixtures (Fig. 1). Proteins in whole cell lysates were digested with trypsin and, after desalting by reversed phase SPE, TiO_2_ beads were added to the SPE eluate. Beads were loaded onto spin columns and washed with buffers of different composition (Fig. 2a), after which peptides were eluted from the TiO_2_ media using an eluent of pH ⩾ 10. Eluted peptides were analyzed by LC–MS/MS. As Fig. 2b illustrates, more than 400 peptides were consistently identified in these TiO_2_ eluates (with just a 45 min LC gradient) regardless of the washing steps applied. Ammonium acetate washes (pH ∼ 6.8) specifically removed non phosphorylated peptides from TiO_2_ beads without resulting in significant phosphopeptide losses. Fig. 2b shows that an enrichment of >85% was achieved in each case.

### 3.2 Factors affecting phosphopeptide enrichment by TiO_2_

**Fig. 3:**
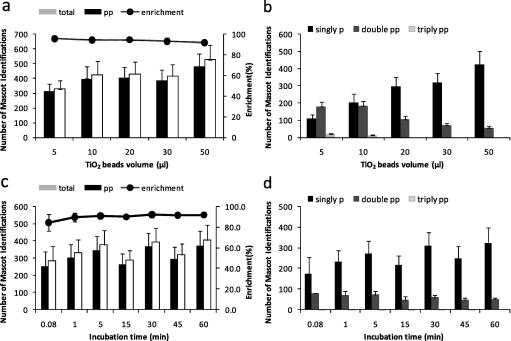
Optimization of volume of TiO_2_ beads and time of incubation. Phosphopeptides were analyzed as in Fig. 1 and identifications returned by Mascot with expectancy scores <0.05 recorded. Percentage of phosphopeptides relative to unphosphorylated peptides was also calculated. (a) Peptide mixtures were incubated with the amounts of TiO_2_ beads shown for 5 min and then analyzed by LC–MS/MS. (b) Number of singly, doubly or triply phosphorylated peptides as a function of TiO_2_ bead volume. (c) Peptide mixtures were incubated with TiO_2_ beads for the indicated times. (d) Number of singly, doubly or triply phosphorylated peptides returned by Mascot as a function of incubation time. Data points are mean ± SEM, *n* = 3 independent experiments.

We next investigated factors that may affect the enrichment of phosphopeptides using TiO_2_. As Fig. 3a illustrates, increasing the ratio of beads to protein amount resulted in an increase in the number of phosphopeptides identified by LC–MS/MS without significantly affecting the enrichment efficiency. Interestingly, the ratio of beads to protein had a dramatic effect on the type of phosphopeptides that were isolated. Thus using 5 μl of beads most of the peptides identified were doubly phosphorylated, whereas singly phosphorylated peptides were predominant when larger bead volumes were used (Fig. 3b). These results are consistent with those published by Li et al. [23].

We also investigated the effect of incubation time on the isolation of phosphopeptides. The results, shown in Fig. 3c, demonstrated that the binding of phosphopeptides to beads was very rapid and that there was no significant difference in the number of phosphopeptides or enrichment efficiency between incubations at 5 s (0.08 min) and 1 min. The number of phosphopeptides isolated increased slightly with longer incubation times reaching a plateau at 30 min. However, we noted that the differences in enrichment between incubations of 5 and 15 min and between 30 and 45 min were not significant (Fig. 3c and d). Incubation time did not have an effect on the distribution of peptides with different numbers of phosphate (Fig. 3d). These data have important implications for the reproducibility and practical implementation of this technique to compare samples and for quantitative analyses, as these time windows allow relatively large number of samples to be handled without introducing variability.

### 3.3 Complexity of the phosphopeptide mixture obtained by TiO_2_

**Fig. 4:**
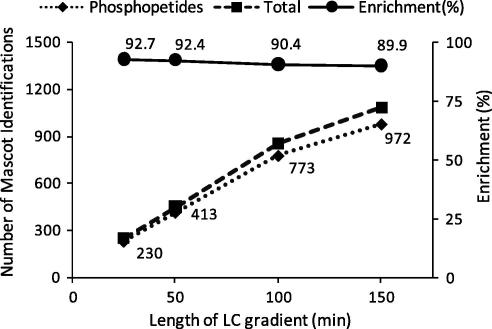
Phosphopeptide identification as a function of LC gradient time. A sample obtained from TiO_2_ extraction was consecutively analyzed using LC gradients of the indicated length. Shown are the number of total peptide and phosphopeptide identifications returned by Mascot with expectancy scores <0.05 and the percentage of phosphopeptides relative to unphosphorylated peptides as a function of LC gradient length. Data is representative of two independent experiments.

**Fig. 5:**
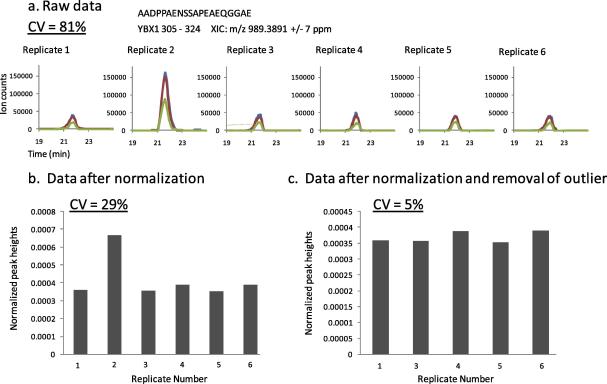
Assessment of precision and the impact of outlier removal from normalized and unnormalized LC–MS quantitative data. Six desalted peptide samples were subjected to phosphopeptide enrichment and LC–MS/MS analysis using our optimized method. A phosphopeptide derived from YBX1 (amino acid position 305–324) at *m*/*z* 989.3891 was selected as an illustrative example of data analysis. (a) XICs of this phosphopeptide in the six replicates. Un-normalized peak heights of these XICs showed a CV of 81%. Blue, red and green traces correspond to the XIC of the first, second and third isotopes, respectively. (b) Peak height intensities values after normalization showing a CV of 29%. (c) Peak height intensities values after normalization and removal of replicate two (this replicate was considered to be an outlier) were quantified with a CV of just 5%.

**Fig. 6:**
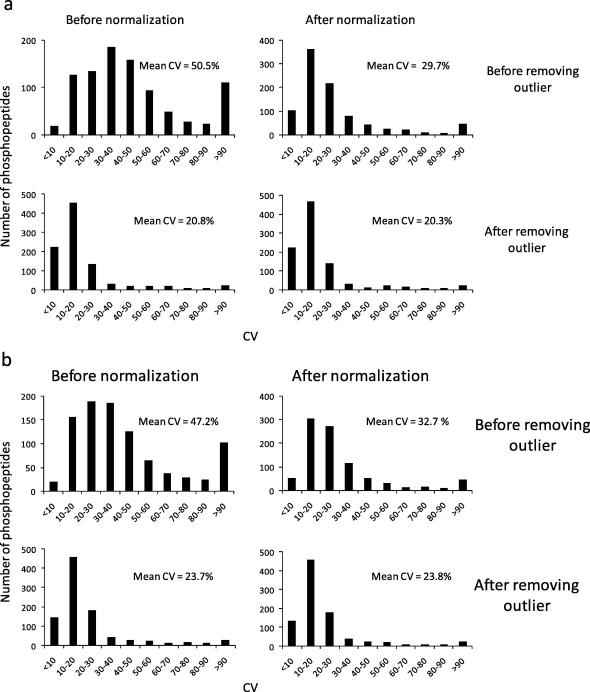
Assessment of precision and impact of data normalization in a large number of phosphopeptides. All the phosphopeptides in the experiment shown in Fig. 5 returned by Mascot with expectancy <0.05 were also quantified and the precision of these measurement assessed by analyzing their variation as in Fig. 5. The charts show the distribution of phosphopeptides with the CV ranges shown. Means of CV of all the phosphopeptides are also shown. Top panels and bottom panels show the distribution of CVs before and after removing the outlier. Left panels and right panels show distribution of CVs before and after normalization, respectively. (a) and (b) show results of peak heights and areas, respectively.

**Fig. 7:**
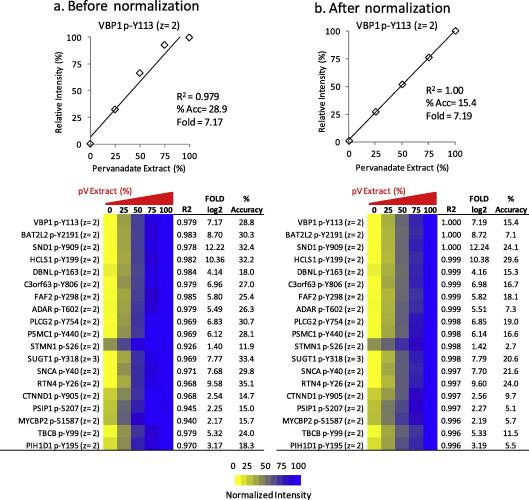
Linearity and accuracy of quantification of phosphopeptides extracted by TiO_2_. P31/Fuj cells were treated with 1 mM sodium pervanadate (pV) or vehicle for 30 min, harvested and lyzed. Five protein mixtures of 0.5 mg of protein each, containing 0%, 25%, 50%, 75% and 100% pV treated extracts balanced with vehicle treated extracts, were processed as in Fig. 1 and analyzed by LC–MS/MS. For the assessment of linearity, relative peptide intensities were correlated with the percentage of pV treated extracts in the mixtures using Pearson’s correlation coefficient (*R*^2^). For the assessment of accuracy, observed values were compared with expected values calculated using linear regression and differences were used to calculate the percentage of accuracy (%Acc). Top panels illustrate the linearity of quantification before (a) and after (b) normalization for the phosphopeptide derived from VBP1 p-Y113 (*z* = 2). *R*^2^, %Acc and fold values in log2 scale are also indicated. Bottom panels show linearity and accuracy data for a larger number of phosphopeptides before (a) and after (b) normalization.

We next investigated the complexity of the phosphopeptide mixture obtained by TiO_2_ enrichment. This question was addressed by analyzing an eluate from TiO_2_ by LC–MS/MS four consecutive times with increasing gradient times (25, 50, 100 and 150 min). The number of unique peptide ions returned by Mascot with expectancy scores lower than 0.05 were recorded as a function of gradient time (Fig. 4). These data revealed that the number of identifications was proportional to gradient time with the 150 min gradient analysis resulting in 972 phosphopeptide ions returned as significant by Mascot. We noted that when using a 150 min gradient the number of identifications started to reach a plateau but had not yet saturated. These results indicate that the number of phosphopeptides in the mixture was probably greater than 1000, even though these analyses were performed with just 500 μg of protein starting material. It is well known that there is only limited overlap of peptide identifications between consecutive LC–MS/MS runs due to undersampling and because low scoring ions from one run may give better scores in other runs [24]. In other words, the stochastic nature of peptide selection and fragmentation by CID leads to variability in *qualitative* data from run to run [24], even though the *quantitative* data may be reproducible (see below). When we combined the data from all the LC–MS/MS runs used to derive the data in Figs. 2–7, a total of 1537 phosphopeptides were returned by Mascot with expectancy scores below 0.05. This number of identifications is quite remarkable given that most of the data (except those in Fig. 4) were obtained from LC–MS/MS runs of just 45 min in length.

### 3.4 Evaluation of TiO_2_ performance for label-free quantification of phosphopeptides

In order to evaluate whether TiO_2_ could be used as an enrichment method for quantitative analyses of phosphorylation, we obtained extracted ion chromatograms (XICs) for all the phosphopeptides identified in the experiment shown in Fig. 2. As an illustrative example of how we performed the analysis, Fig. 5 shows the evaluation of one phosphopeptide at *m*/*z* 989.3891 matched to Nuclease sensitive element-binding protein 1 (gene name: YBX1). The intensities of the XICs for this phosphopeptide obtained from 6 independent TiO_2_ extractions showed a CV of 81% (Fig. 5a). Normalization of the intensities (peak heights) of the XICs resulted in an increase of the precision of the measurement. Indeed as Fig. 5b illustrates, the CV was just 29% after data normalization. The precision was further increased to a CV of only 5% when the measurement from replicate two was removed (Fig. 5c), as this replicate was an obvious outlier (Fig. 5a and b).

We next sought to evaluate the precision of quantification of the technique on a larger number of phosphopeptides. Combining the data shown in Fig. 2 led to a total of 937 phosphopeptide identifications with Mascot expectancy scores below 0.05. We constructed XICs for all of these peptides and calculated their intensities (peak heights and areas) across six replicates (independent extractions). The mean CV of the intensities obtained from the raw data was about 50% on average (Fig. 6a). After normalization, the precision improved to a CV of ∼30% on average (Fig. 6a) and to just 20% after removing replicate two from the analysis (Fig. 6d), which as discussed above was found to be an outlier. It is interesting to note that data normalization (Fig. 6b) can compensate for the presence of outliers to a great extent, albeit not completely, as removal of the outlier resulted in greater precision of measurement (Fig. 6c) than normalization by itself (Fig. 6b). These data highlight the importance of performing replicate measurements to enhance data quality. We also assessed the precision of the method using peak area as a measurement of intensity (Fig. 6b). The results obtained from the analysis based on peak heights and areas were similar, although the precision of the measurement using peak heights seemed to be slightly greater than when the analysis was performed taking peak areas as the quantitative readout (compare Fig. 6a with Fig. 6b).

The data in Fig. 6 indicated that, after normalization, phosphopeptides extracted by TiO_2_ can be analyzed with good precision. However, accuracy of quantification is related to the linearity of detection rather than to the reproducibility of measurement [15]. Therefore, we also determined the linearity of quantification of phosphopeptides present in TiO_2_ eluates using our recently published method to assess linearity of quantification in a global scale [15]. To this end, cells were treated with pervanadate (pV), a protein tyrosine phosphatase inhibitor, which, as a knock-on effect, also induces phosphorylation on serines and threonines [12]. Cells treated with pV were mixed at different proportions with cells left untreated before phosphopeptide extraction and LC–MS/MS analysis. Fig. 7 shows the linearity of quantification of a peptide derived from VBP1 phosphorylated at Y113. Although the correlation coefficient of the data before normalization was adequate (*R*^2^ = 0.979, Fig. 7a top panel), normalization procedures led to a greater linearity of quantification for this phosphopeptide (*R*^2^ = 1.00, Fig. 7b top panel). Analysis of linearity for a larger number of phosphopeptides also revealed that normalized data showed larger correlation coefficients than the same data before normalization (compare bottom panels of Fig. 7a and b).

The accuracy of the measurements was also calculated for each phosphopeptide quantified. This entailed calculating the deviation between the observed intensities and the theoretical intensities obtained from the linear regression function [15]. This analysis revealed that most phosphopeptides could be quantified with accuracy deviations (%Acc) < 30% and that normalization improved accuracy of quantification (examples are shown in Fig. 7 lower panels).

Taken together, these data indicate that MS signals from phosphopeptides present in TiO_2_ eluates are proportional to the concentration of the phosphopeptide from which they originated in biological samples and that these can therefore be quantified with good accuracy (Fig. 7) and precision (Figs. 5 and 6).

## 4 Discussion

Phosphorylation is a protein post-translational modification with important regulatory functions; consequently, there has been a large body of work dedicated to the development of methods for its investigation. An important consideration in phosphoproteomics is that phosphorylation is most commonly found at sub stoichiometric levels on proteins. In addition, although modern mass spectrometers can detect peptides at low attomole amounts in samples, even state-of-the-art instruments are still limited by their dynamic range of detection. This means that, in complex mixtures, abundant non-modified peptides are detected by MS/MS and LC–MS/MS in preference to low abundance phosphopeptides. Therefore, techniques to enrich samples for phosphopeptides prior to MS analysis were required for the field of phosphoproteomics to advance. These efforts led to the development of affinity purification methods based on antibodies (chiefly against anti-phosphotyrosine), immobilized metal affinity chromatography (IMAC) and TiO_2_. These methods have been shown to be robust for phosphopeptide isolation and applicable to complex mixtures [13–25,31]. However, the data on the utility of current techniques of phosphopeptide enrichment for quantitative analysis is very limited. We have previously shown that IMAC extraction is compatible with large-scale label-free quantitative analyses [15]. However, limitations were that large volumes of IMAC beads (∼300 μl) were needed for the technique to be quantitative and enrichment efficiency was around 30–50% at best. Therefore, we decided to investigate TiO_2_ as the basis of a quantitative technique for label-free quantitative phosphoproteomics. An attractive feature of TiO_2_ is that it has greater affinity for phosphopeptides than IMAC; therefore less bead volume is required, thus permitting the use of less sample volume, making the experiment easier to handle and increasing the throughput. Another advantage of the use of TiO_2_ is that the extraction efficiency is greater than that offered by IMAC (at least in our hands).

Although many reports have shown that TiO_2_ is an efficient method to enrich for phosphopeptides, there are no reports documenting the performance of this technique for label-free quantitative analysis of phosphorylation. Thus as a novel aspect of the present study, we have shown that TiO_2_ allows reproducible phosphopeptide enrichment from total cell digests (Fig. 2). Individual phosphopeptides could be quantified with good precision (Figs. 5 and 6) and linearity/accuracy (Fig. 7) without the need to use chemical or metabolic labeling. These results indicate that, although there is variability in ion yields of peptides with different sequences and the intensity of one phosphopeptide cannot be correlated with the concentration of phosphopeptides of unrelated sequences, phosphopeptide signals obtained by LC–MS from TiO_2_ enriched samples were proportional to their own concentration in biological samples. Introduction of robust normalization strategies and running of replicates to detect outliers (which, if present, should be removed after normalization) improved the precision of the technique (Fig. 5).

The purpose of the present study was to investigate and document the performance of TiO_2_ for label-free quantitative phosphoproteomics rather than to identify a large number of phosphopeptides. We therefore used relatively short gradient times (45 min) and did not perform an initial separation step to increase peak capacity. We also used only 500 μg of protein (obtained from just about two million cells) per experimental condition. This amount of material is relatively small and the analysis time is short when compared with published large scale analyses of the phosphoproteome, which normally entail the use of several milligrams of protein and several days of LC–MS/MS analysis time per sample [16–32]. Nevertheless, under the conditions used in the present study, hundreds of phosphopeptides could be detected when the data of several LC–MS/MS runs were pooled (Fig. 4 and data not shown). The number of identifications had not saturated at high gradient lengths (Fig. 4), suggesting that several thousand phosphopeptides were present in TiO_2_ eluates obtained from just 500 μg of protein material.

In conclusion, appropriately controlled TiO_2_-based extraction methods permit the extraction of phosphopeptides from complex peptide mixtures in a reproducible and efficient fashion. Several hundreds, perhaps even thousands of phosphopeptides, can be obtained with this method from relatively small amounts of material. MS analysis of phosphopeptides in TiO_2_ eluates allows the generation of high-content data and the quantification of phosphorylation with good precision and linearity/accuracy. Since these analyses do not require the use of chemical or metabolic labeling, the technique should have broad applicability in studies aimed at quantifying signaling in a wide array of biological samples including those derived from primary and clinical material.
